# The Anatomy and Philosophy of Expression

**Published:** 1845-01

**Authors:** 


### THE ANATOMY AND PHILOSOPHY OF EXPRESSION, AS CONNECTED WITH THE FINE ARTS. By Sir *Charles Bell*, K.H. &c. 3rd. Edition, enlarged. 8vo. pp. 265. London: Murray, 1844.

THIS work has a peculiar, and even melancholy, interest in more respects than one. It is the first and the last of the gifted author's published writings. It was originally composed while he was yet a student, "before"—as he gracefully says in the dedication to his brother—"the serious pursuits of life began;" and he was engaged, we believe, in revising the present edition for the press, when he was seized with his fatal illness at Hallow Park, in Worcestershire, on the 29th of April, 1842.

*Charles Bell* was a man of a truly original cast of mind. From his youth, he had learned to think for himself; and his thoughts had early been turned to the study of the Nervous System. Being an accomplished draughtsman, and having naturally a lively taste for the fine arts, he was in the habit of occupying his leisure hours with portraying, alike with his pencil and his pen, the outward and visible features of the various passions that agitate the human breast. The taste for such pursuits never left him; the very day before his death, he was engaged in his favourite amusement. He much mistook, we have often thought, the part which Nature designed him for, when he applied himself to the active pursuit of practical surgery. The bias of his mind lay in quite an opposite direction. He was too contemplative a character, and, moreover, his feelings were far too sensitive, for many of the every-day duties of a hospital surgeon. He had a dislike for all cutting operations, and an aversion from inflicting pain even on the lower animals. In spite of all his exertions, he never could have become an *Astley Cooper* or a *Dupuytren*: he was not made of the same stuff as these men. Each had his reward. The leading surgeon of the French, as well as he of the English, metropolis enjoyed a boundless reputation during his life, and left an enormous fortune behind him. The fame of both stood high for many years; but now, if we mistake not, it is on the wane; and, ere long, these once great surgeons will cease to be regarded as shining lights in the professional hemisphere. Not so with *Bell*. But partially appreciated by his cotemporaries, his reputation will unquestionably increase with increasing years; and posterity, we doubt not, will rank his name with those of *Harvey* and *John Hunter*.

Sir Charles had early caught a glimpse of that great discovery which shed so bright a lustre over his maturer years; and certainly not the least interesting feature of these Essays is, that they clearly contain the germ of this discovery, and shew us some of the steps by which he was led to its attainment. It is a vulgar error to suppose that the finding out of any great and comprehensive truth was ever made by what is generally termed "a lucky thought," or without the previous discipline of long and deep reflection on the subject. There was no exception to this remark in the case of our lamented author. At the very threshold of his professional career, he saw and accurately described the wide extent and the complicated relations of the function of Respiration; pointing out how distant and seemingly unconnected parts of the body are intimately bound and associated together, in the performance of this vital act, and of the numerous accessory phenomena that are dependent upon it. With no less physiological acumen than with refined artistic taste, he perceived that it was not the lungs and the muscles of the chest *alone* that are engaged in the carrying on of the process of breathing, under the excitement of any passion; but that the muscles of the face, throat, neck, shoulders and trunk are *then* all, more or less, immediately implicated. The great painters and sculptors of Greece had fully understood the truth of this corporeal complex association, by studying the undressed figures of their countrymen in their gymnastic games, and other exhibitions of agility, strength, and passion. They knew not indeed the cause of those combined physical appearances; but, by closely observing Nature, they had learned to represent them with marvellous fidelity in their pictorial and sculptural designs. It remained for Charles Bell to discover the *why* of the sympathetic union of so many parts in the production of one effect; to find out the clue of a complex and seemingly-entangled web; to educe harmony from seeming discord, and the most orderly design from seeming confusion. Few medical men are, we believe, aware of the simple yet comprehensive grandeur of his discovery of the respiratory system of nerves; they may know its anatomical details, but they have not studied it in a physiological or an artistic point of view; nor have they even learned to appreciate its intimate relations with many of the most common acts of the body, such as crying, laughing, weeping and so forth. Each of these acts is nothing but a natural and spontaneous effort of the system to restore, to normal quietude, a disturbed state of the breathing and of the circulation of the blood; and all the visible phenomena or outward expressions of the Emotions, which give rise to them, are more or less directly connected with this instinctive effort—this *vis conservatrix naturæ.*

The Breast is the part of the body that is instinctively referred to as the *seat* of the passions; and the common language of every people gives currency and sanction to this idea. And yet we know that they are only mental acts or varying conditions of the immaterial mind, and cannot therefore be seated in the body, however much they may influence or be influenced by it. The effect is thus substituted for the cause; the bodily feeling for the mental act. So intimate however is the connection between these two states that, if the bodily sensation is in any way induced independently of the operation of the mind, the cognate and corresponding sentiment, or something very much akin to it, will at once be experienced. For example, do we not daily observe that the tremor of the limbs, the coldness of the skin, the chattering of the teeth and rapid throbbing of the heart, in the common act of febrile shivering, are invariably accompanied with the feelings of trepidation and fear?—and will not the mere effort of clenching the fist, holding the breath, and steadily fixing the eye upon one object, induce—almost in spite of our efforts to the contrary—a sentiment of indignant resolution in our breasts? How admirably has Shakespeare illustrated the force of this alliance in the following well-known passage, in which Henry V. strives to inflame the zeal and courage of his soldiers!

> "But when the blast of war blows in our ears,
> Then imitate the action of the tiger:

Stiffen the sinews, summon up the blood,
Disguise fair Nature with hard-favour'd rage !
Then lend the eye a terrible aspect;
Let it pry thro' the portage of the head,
Like the brass cannon : let the brow o'erwhelm it,
As fearfully as doth a galled rock
O'er-hang and jutty his confounded base,
Swill'd with the wild and wastful ocean.
Now set the teeth, and stretch the nostril wide;
Hold hard the breath, and bend up every spirit
To his full height."

Sir Charles ingeniously attempts to explain, or at least illustrate, this connexion between certain bodily states and the development of certain mental emotions, by directing our attention to something analogous to it in the exercise of our sensual perceptions. The following passage will best convey to our readers the train of reasoning whereby he endeavours to shew, that what the Eye, the Ear, or the Tongue is to the mind, as exciting those ideas which have been appointed to correspond with the qualities of the material world, the organs of the Breast are to the development of our affections.

" By emotions are meant certain changes or affections of the mind, as grief, joy, astonishment. That such states or conditions of the mind should in any degree pertain to the body, may not, perhaps, be willingly admitted; unless we take along with us that the ideas of sense, as light, sound, or taste, are generated by the organs of the senses, and not by any thing received and conveyed by them to the sensorium. It is ascertained that the different organs of the senses can be exercised, and give rise to sensation and perception, when there is no corresponding outward impression; and the ideas thus excited are according to the organ struck or agitated: that is, the same impression, conveyed to different organs of sense, will give rise to a variety of sensations; as light, when the eye is struck; sound, when the ear is struck; and so on with the other organs; the sensation corresponding with the organ which is exercised, and not with the cause of the impression. A needle passed through the retina, the organ of vision, will produce the sensation of a spark of fire, not of sharpness or pain; and the same needle, if applied to the papillæ of the tongue, will give rise to the sense of taste; while if it prick the skin, pain will follow. This law of the senses is arbitrarily or divinely ordered; it might have been otherwise. Accordingly, when we observe that the organs of the senses operate in producing specific ideas, independently of their own peculiar exciting causes, we can comprehend better how other organs of the body may have a relation established with the mind, and a control over it, without reference to outward impressions." 85.

Without pursuing this subject, which touches on the limits of metaphysical enquiry, let us now endeavour to illustrate, by a few examples, the expression or corporeal manifestation of certain strong mental emotions, with the view of pointing out the intimate alliance between the development of such expression, and a disturbed state of the heart and the machinery of respiration: and first of *Fear*. What says the sacred penman, in the account of the solemn vision that he saw, when deep sleep falleth on men? " Fear came upon me and trembling, which made all my bones to shake. Then a spirit passed before my face; the hair of my flesh stood up. It stood still, but I could not discern the form thereof; an image was before mine eyes; there was silence, and I heard a voice."

The chief features in this impressive description are reproduced in these lines of *Virgil*:

"Mihi frigidus horror
Membra quatit, gelidusque coit formidine sanguis."
* * * * * *
"Arrectæque horrore comæ, et vox faucibus hæsit."

In other passages, the stiffened gaze and the relaxed powerlessness of limb are well described.

"At juveni oranti subitus tremor occupat artus,
Diriguere oculi;"
"Illi membra novus solvit formidine torpor."

*Lucretius* has graphically introduced many of the leading characteristics of terror in the following passage:

—— "Ubi vehementi magis est commota metu mens,
Consentire animam totam per membra videmus:
Sudores itaque et pallorem existere toto
Corpore, et infringi linguam, vocemque oboriri,
Caligare oculos, sonere aureis, succidere artus."

Compare with these several descriptions the following one by our author: it will serve to render the portrait more complete.

"We can readily conceive why a man stands with eyes intently fixed on the object of his fears, the eyebrows elevated to the utmost, and the eye largely uncovered; or why, with hesitating and bewildered steps, his eyes are rapidly and wildly in search of something. In this, we only perceive the intent application of his mind to the object of his apprehensions—its direct influence on the outward organ. But observe him further: there is a spasm on his breast, he cannot breathe freely, the chest is elevated, the muscles of his neck and shoulders are in action, his breathing is short and rapid, there is a gasping and a convulsive motion of his lips, a tremor on his hollow cheek, a gulping and catching of his throat; and why does his heart knock at his ribs, while yet there is no force of circulation?—for his lips and cheeks are ashy pale." 88.

In the agony of Grief, too, how strongly are all the organs of breathing affected! The respiration is low and infrequent, but every now and then broken by sobs and long-drawn sighs; the neck and throat are convulsed with struggling, and their veins are full almost to bursting; the face is deadly pale, while slight quivering motions pass from time to time over it; the lips are swollen and trembling;* tears gush forth in torrents from the eyes; the hands are icy-cold; the limbs are nerveless and relaxed, and the poor sufferer sinks almost unconscious upon the ground. Now, if we attend to the manner in which these corporeal phenomena arise and succeed each other, it will be found that the primary effect of the vehement emotion is an immediate suspension of the act of breathing, followed by convulsive deep inspirations, and violent rapid expirations. These efforts are designed by Nature to relieve the congestion of the heart, and give

* "The action of the Orbicular muscle of the lips is, indeed, the most characteristic of agony of mind, and of all those passions which partake of sentiment; in grief, in vexation of spirit, in weeping, it modifies the effect of the muscles of animal expression, and produces human character."

freer play to the pulmonary circulation that has become in some degree obstructed, in consequence of the embarrassed state of the breathing. Who is there that has not experienced that sense of oppressive fulness, nay even of sickening pain, in the cardiac region, when the mind is bowed down by some overwhelming affliction? And who has not felt the relief that is given thereto by the heaving sob, and the bursting tear?

> "The grief that does not speak,
> Whispers the o'erfraught heart, and bids it break."

Ovid has given a fine description of the stupefaction which sometimes characterises intense grief—"the lethargy of woe:"

> "Obmutuit illa dolore;
> Et pariter vocem, lacrymasque introrsus obortas
> Devorat ipse dolor; duroque simillima saxo
> Torpet; et adversâ figit modo lumina terrâ:
> Interdum torvos sustollit ad æthera vultus;
> Nunc positi spectat vultum, nunc vulnera nati."—*Met. Lib.* xiii.

How different is the expression of Laughter on the one hand, or of Rage and severe Pain on the other, from that of the depressing passions!

"Observe the condition of a man convulsed with laughter, and consider what are the organs or system of parts affected. He draws a full breath, and throws it out in interrupted, short, and audible cachinnations; the muscles of his throat, neck, and chest, are agitated; the diaphragm is especially convulsed. He holds his sides, and, from the violent agitation, he is incapable of a voluntary act.

"It is impossible to avoid the conclusion, that it is the respiratory organs and their muscles which are affected during the paroxysm of laughter. Physiologists, in all former times, attributed the line of sympathetic relations which draw these remote parts into action, to a nerve called the sympathetic. But I have proved, that there is a machinery altogether distinct; and that the expression, not only of this, but of all the other passions, arises from that system of nerves, which, from their great office, I have called *respiratory*." 147.

The effects of Rage are best portrayed in the savage animal.

"When the tiger or wolf," says our author, "is struck by the keeper, and suddenly roused to ferocity and activity, the character is seen not only in the glare of the eyes, the retraction of the lips, and the harsh sound of the breath as it is forcibly drawn through the confined throat, but every muscle is in tension, and the limbs in an attitude of strained exertion, prepared to spring. In this condition of high animal excitement, observe the manner in which the chest is kept distended and raised; the inspiration is quick, the expiration slow; and, as the keeper strikes the jaw, there is at the same instant a start into exertion, and the breath rapidly drawn in." 190.

In the human subject, this passion is characterised by the clenching of the teeth, the sparkling of the eyes, the inflated state of the nostrils, the knitting of the eyebrows, the holding of the breath, the closing of the fist, and by the tension of the muscles in every part of the body and limbs. The expression is not confined to one part or feature; but it pervades, and to the practised eye is visible in, the whole frame.

The Latin poets are, on the whole, not very successful in describing the effects of Rage. Outcries and bellowings constitute almost invariably one of the most prominent features of their portraits. *Virgil* has surely carried poetic license rather too far when he makes sparks of fire to burst from the mouth, as well as from the eye:

> " His agitur furiis, totoque ardentis ab ore
> Scintillæ absistunt; oculis micat acribus ignis.
> Mugitus veluti cum prima in prælia taurus
> Terrificos ciet, atque irasci in cornua tentat!"

In intense bodily Pain, as in Rage, almost every part of the body is in a state of convulsive excitement. The jaws are fixed, and the teeth grind; the lips are drawn laterally and the nostrils dilated; the eyes are largely uncovered and the eyebrows raised; the face is turgid with blood, and the veins of the temple and forehead distended; the breath being checked, and the descent of blood from the head impeded by the agony of the chest, the cutaneous muscle of the neck acts powerfully and draws down the angles of the mouth; the arms are outstretched, and the limbs are either convulsively contracted, or rigidly extended. One of the most striking features of the agony of Pain is a fixed and distended elevation of the chest. The object of this will at once be apparent, when we call to mind that the muscles of the arms—which are then, it is well known, instinctively thrown into the most violent action—arise from the chest. It is therefore necessary that their points of origin be immoveable, in order that their other extremities, or insertions into the limbs, may act with the greatest amount of power: now this can only be done by the chest being kept continually fixed and expanded.

At the same time we must remember that, as long as the chest remains in this condition, no loud or prolonged cries can be uttered. "Hence," says our most ingenious author, "that most terrible silence in human conflict, when the outcry of terror or pain is stifled in exertion; for, during the struggle with the arms, the chest must be either expanded or in the act of rising; and therefore the voice, which consists in the expulsion of the breath by the falling or compression of the chest, is suppressed, and the muscles, which perform the office of raising and distending the chest, act in aid of the muscles of the arms."

The truth of these observations is beautifully illustrated by a reference to the famous group of "Laocoon and his Sons," now in the Vatican. Every one knows that this statue is justly considered to be one of the greatest achievements of Grecian Art. In the second book of the Æneid, *Virgil* has described, with his usual felicity, the terrible fate of the devoted priest. Already had the messengers of divine wrath encircled him with their inextricable folds:

> " Ille simul manibus tendit divellere nodos,
> Perfusus sanie vittas atroque veneno;
> Clamores simul horrendos ad sidera tollit:
> Qualis mugitus, fugit quum saucius aram
> Taurus, et incertam excussit cervice securim."

Mr. *Payne Knight* has, with no little acumen, censured this description in the following passage from his Essay on Taste.

" It is not with the agonies of a man, writhing in the pangs of death, that we sympathise, on beholding the celebrated group of Laocoon and his sons; for such sympathies can only be painful and disgusting: but it is with the energy and fortitude of mind which those agonies call into action and display. For though every feature and every muscle is convulsed, and every nerve contracted, yet the breast is expanded and the throat compressed, to shew that he suffers in silence. I therefore still maintain, in spite of the blind and indiscriminate admiration, which pedantry always shews for every thing which bears the stamp of high authority, that Virgil has debased the character, and robbed it of all its sublimity and grandeur of expression by making Laocoon *roar like a bull;* and I think that I may safely affirm, that if any writer of tragedy were to make any one personage of his drama to roar out in the same manner, on being mortally wounded, the whole audience would burst into laughter, how pathetic soever the incidents might be that accompanied it. Homer has been so sensible of this, that of the vast number and variety of deaths, which he has described, he has never made a single Greek cry out on receiving a mortal wound." 192.

Sir *Charles* approves of this criticism, so far as the artist is praised and the poet is blamed; but points out, at the same time, a fallacy in the critic's views, and shews that he has somewhat mistaken the aim of the artist. After admitting that he himself, upon examining the statue at Rome, was convinced that 'Laocoon' is represented by the sculptor as suffering *in silence,** he proceeds to observe:

"The artist did not mean to express 'energy and fortitude of mind,' or, by 'expanding the breast and compressing the throat, to shew that he suffers in silence.' His design was to represent corporeal exertion, the attitude and struggles of the body and of the arms. The throat is inflated, the chest straining, to give power to the muscles of the arms, while the slightly parted lips shew that no breath escapes; or, at most, a low and hollow groan. He could not roar like a bull—he had not the power to push his breath out in the very moment of the great exertion of his arms to untwist the serpent which is coiled around him. It is a mistake to suppose that the suppressed voice, and the consent of the features with the exertion of the frame, proceed from an effort of the mind to sustain his pain in dignified silence; for this condition of the arms, chest, and face, are necessary parts of one action.

"The instant that the chest is depressed to vociferate or bellow, the muscles arising from the ribs and inserted into the arm-bones must be relaxed, and the exertion of the arms becomes feeble. Again, in speaking or exclaiming, a consent runs through all the respiratory muscles; those of the mouth and throat combine with those which move the chest. Had the sculptor represented Laocoon as if the sound flowed from his open mouth, there would have been a

---

* *Lord Byron,* we may presume, was of the same opinion; otherwise, the allusion to the sufferer's *patience,* in his celebrated lines, would not apply with truth:

"Or, turning to the Vatican, go see
Laocoon's torture dignifying pain—
A father's love and mortal's agony
With an immortal's patience blending:"

In his "Prometheus," also, he expresses a similar thought:

"A silent suffering and intense;
The rock, the vulture, and the chain,
All that the proud can feel of pain,
The agony they do not shew,
The *suffocating* sense of woe,
Which speaks but in its loneliness."

strange inconsistency with the elevated condition of his breast. Neither is it correct to suppose it possible that a man struck down with a mortal wound, and rolling in the dust, like Homer's ill-fated heroes, can roar out like a bull. A mortal wound has an immediate influence on these vital parts and respiratory organs, and the attemp to cry aloud would end in a feeble wail or groan." 193.

The latter part of this beautiful quotation naturally enough leads us to notice the varying expression of violent Death—as revealed not only in the features of the face, but also in those of the body generally—according to the nature of the struggles that have preceded the mortal agony. Let us take a few examples. How different are the outward marks of death induced by suffocation, from those in the body of one who has died from loss of blood! *Shakespeare* has described the first:

> " But, see, his face is black and full of blood;
> His eyeballs further out than when he lived,
> Staring full ghastly like a strangled man;
> His hair upreared, his nostrils stretched with struggling;
> His hands abroad displayed as one that grasped
> And tugged for life, and was by strength subdued.
> Look on the sheets; his hair, you see, is sticking;
> His well-proportioned beard made rough and rugged,
> Like to the Summer's corn by tempest lodged."
>
> *Henry VI. Part II.*

Contrast this horribly true picture of death from strangling, with that of Euryalus, who has fallen, pierced through the chest by his Volscian foe:

> " Viribus ensis adactus
> Transadigit costas, et candida pectora rumpit.
> Volvitur Euryalus letho, pulchrosque per artus
> It cruor, inque humeros cervix collapsa recumbit:
> Purpurens veluti quum flos, succisus aratro,
> Languescit moriens; lassove papavera collo
> Demisere caput, pluvia quum forte gravantur."—*Æneid*, ix. 433.

Admirably does Sir *Charles* describe the convulsive agonies of the whole machinery of respiration, that precede death from Hæmorrhage, in the following words:

" The 'Dying Gladiator' is one of those masterpieces of antiquity which exhibits a knowledge of anatomy and of man's nature. He is not resting; he is not falling; but in the position of one wounded in the chest and seeking relief in that anxious and oppressed breathing which attends a mortal wound with loss of blood. He seeks support to his arms, not to rest them or to sustain the body, but to fix them, that their action may be transferred to the chest, and thus assist the labouring respiration. The nature of his sufferings leads to this attitude. In a man expiring from loss of blood, as the vital stream flows, the heart and lungs have the same painful feeling of want, which is produced by obstruction to the breathing. As the blood is draining from him, he pants, and looks wild, and the chest heaves convulsively. And so the ancient artist has placed this statue in the posture of one who suffers the extremity of difficult respiration. The fixed condition of the shoulders, as he sustains his sinking body, shews that the powerful muscles, common to the ribs and arms, have their action concentrated to the struggling chest. In the same way does a man afflicted with asthma rest his hands or his elbows upon a table, stooping forwards, that the shoulders may become fixed points; the muscles of the arm and shoulder then act as muscles of respiration, and aid in the motion of the chest, during the heaving and anxiety which belong to the disease." 195.

This description is rendered the more affecting, by being accompanied with a beautiful drawing of this masterpiece of Ctesilaus, representing (as *Winkelmann* has justly said) "a wounded man dying, who perfectly expressed what there remained of life in him."

There is an interesting section in one of these Essays, in which our author suggests some strictures on the pictorial representation of "Demoniacal possession," or Convulsionary attacks, by those great masters of their art, *Domenichino* and *Raffaelle.* One of the frescoes, by the former, in the Convent of the Grotto Ferrata near Rome, represents Saint Nilus in the act of miraculously curing a lad that is possessed with a devil. "Convulsions have seized him; he is rigidly bent back; the lower limbs spasmodically extended, so that his toes only rest on the ground; the eyes are distorted, and the pupils turned up under the eyelids. This would be the position of Opisthotonos, were not the hands spread abroad, the palms and fingers open, and the jaw fallen. Had the representation been perfectly true to Nature, the jaws would have been clenched, and the teeth grinding. But then the miracle could not have been represented; for one, under the direction of the Saint, has the finger of his left hand in the boy's mouth, and the other holds a vessel of oil, with which the tongue is to be touched."

In the famous Cartoon by *Raffaelle*, of "The death of Ananias," the effect would have been more impressive—if it was really the painter's intention to excite horror—had there been greater truth in the convulsions of the dying sinner, instead of a mere twisting of the body.

"In the same painter's great picture of the Transfiguration, in the Vatican, there is a lad possessed, and in convulsions. I hope I am not insensible to the beauties of that picture, nor presumptuous in saying that the figure is not natural. A physician would conclude that this youth was feigning. He is, I presume, convulsed; he is stiffened with contractions, and his eyes turned in their sockets. But no child was ever so affected. In real convulsions, the extensor muscles yield to the more powerful contractions of the flexor muscles; whereas, in the picture, the lad extends his arms; and the fingers of the left hand are stretched unnaturally backwards. Nor do the lower extremities correspond with truth; he stands firm; the eyes are not natural; they should have been turned more inwards, as looking into the head, and partially buried under the forehead. The mouth, too, is open, which is quite at variance with the general condition, and without the apology which Domenichino had. The muscles of the arms are exaggerated to a degree which Michael Angelo never attempted; and still it is the extensors and supinators, and not the flexors, which are thus prominent." 161.

However strictly true such strictures may be, in a pathological point of view, our author candidly admits that, "were the painter to represent every circumstance (of such an attack) faithfully, the effect might be too painful; something must be left to his taste and imagination."

With two or three remarks on the Expression communicated by the *eye* in certain states of the system, we shall close our notice of this charming volume—charming alike by its physiological expositions, its artistic illustrations, and its classical allusions and extracts.

"The *orbicularis* muscle of the eyelids acts powerfully in certain kinds of expression. In laughing and crying, the outer circle of this muscle, as it contracts, gathers up the skin about the eye; and at the same time it compresses the eyeball. A new interest is given to the subject when we inquire into the object of that compression. It has a distinct relation to the circulation of the blood within the eye. During every violent act of expiration, whether in hearty laughter, weeping, coughing, or sneezing, the eyeball is firmly compressed by the fibres of the orbicularis; and this is a provision for supporting and defending the vascular system of the interior of the eye from a retrograde impulse communicated to the blood in the veins at that time. When we contract the chest, and expel the air, there is a retardation of the blood in the veins of the neck and head; and in the more powerful acts of expulsion, the blood not only distends the vessels, but is even regurgitated into the minute branches. Were the eye not properly compressed at that time, and a resistance given to the shock, irreparable injury might be inflicted on the delicate textures of the interior of the eye. Hence we see a reason for the closed state of the eyelids, and wrinkling of the surrounding skin, and twinkling of the eye, in hearty laughter." 106.

Whenever there is an actual or impending stupefaction of the system, the voluntary or *recti* muscles of the eyeball resign their action to the *oblique* muscles; the effect of which is, that the eye revolves upwards, and the white of the ball is exposed; at the same time, the *levator palpebræ superioris* yields, in sympathy with the oblique muscles, to the action of the *orbicularis* which closes the eye, and the eyelid drops. It is the struggle of the drunkard to resist, with his half-conscious efforts, the rapid turning-up of the eye, and to preserve it under the control of the voluntary muscles, that makes him see things distorted or double; and strive, by arching his eyebrows, to prevent the dropping of the upper eyelid. The puzzled appearance which this gives rise to, along with the relaxation of the lower part of the face, and the slight paralytic obliquity of the mouth, completes the degrading expression. In another passage our author beautifully observes: "There is sometimes in death a fearful agony in the eye; but we have said, that it is consolatory to know that this does not indicate suffering, but increasing insensibility. The pupils are turned upwards and inwards. This is especially observed in those who are expiring from loss of blood. It is the *strabismus patheticus orantium* of Boerhaave. Sauvages observes on this rolling up of the eyeball, in dying children,—'Vulgo aiunt hos tenellos suam patriam respicere.' 'The vulgar say, that these little ones are looking to their native home.'"

